# Supplementary material for: Investigating self-reported efficacy of lifestyle medicine approaches to tackle erectile dysfunction: a cross-sectional eSurvey based study
Source: BMC Urol. 2023 Feb 6;23:15. doi: 10.1186/s12894-023-01180-2 (PMC9901095; doi:10.1186/s12894-023-01180-2)
Supplement: Supplementary file 1 — Additional file 1. Copy of community-facing elecronic survey. [file 12894_2023_1180_MOESM1_ESM.pdf]

Barriers and Drivers for the Provision of Lifestyle Medicine approaches to Tackle Erectile Dysfunction

(community facing)

Start of Block: Default Question Block

Q1
Imperial College London Self-Care Academic Research Unit (SCARU) is collaborating with Regimen (a smartphone app that offers a CE-certified digital program for treating erectile dysfunction sustainably, as a long-term solution to erectile dysfunction) to gauge the knowledge, attitude & perceptions regarding erectile dysfunction (ED) interventions & to better understand how lifestyle medicine approaches can be used to tackle ED in the community setting.

Please take a moment to review the Participant Information Sheet of our study which, also has ethical approval in place, and consider taking part in this 5-10 min eSurvey. Please feel free to disseminate the study information & link to your personal contacts.

Q2 Do you consent to participate in this survey?

○ Yes (1)

○ No (2)

Skip To: End of Block If Do you consent to participate in this survey? = No

---

© Imperial College of Science, Technology and Medicine

Q3 How would you describe your gender?

- ○ Male (1)
- ○ Female (2)
- ○ Other (please specify) (5) ____
- ○ Prefer not to say (6)

Q4 Have you ever had an experience when you could not get an erection?

- ○ Yes (1)
- ○ No (2)

Q5
Have you had issues in getting an erection over an extended period of time (3-6 months)?

- ○ Never (7)
- ○ Rarely (8)
- ○ Sometimes (11)
- ○ Frequently (9)
- ○ Often (10)

Q6 Over the last 3-6 months, rate your confidence to **get** an erection:

- ○ Very low (1)
- ○ Low (2)
- ○ Moderate (3)
- ○ High (4)
- ○ Very high (5)

Q7 Over the last 3-6 months, rate your confidence to **maintain** an erection:

- ○ Very low (1)
- ○ Low (2)
- ○ Moderate (3)
- ○ High (4)
- ○ Very high (5)

Q8 How important is a firm & lasting erection to your mental & physical wellbeing & quality of life?

- ○ Very unimportant (1)
- ○ Unimportant (2)
- ○ Important (3)
- ○ Very important (4)

Q9 If you had trouble with getting or maintaining an erection, would you feel comfortable discussing this with:

	Yes (1)	No (2)
Family (1)	○	○
Friends (6)	○	○
Partner/spouse (8)	○	○
GP/ doctor (2)	○	○
Pharmacists (4)	○	○
Sexual health clinic (5)	○	○
Support groups (e.g online forums) (7)	○	○
Chatbot / Avatar (9)	○	○

Display This Question:

If Have you had issues in getting an erection over an extended period of time (3-6 months)? != Never

© Imperial College of Science, Technology and Medicine

Q10 Where did you get information about resolving the issue?

- ☐ GP/ medical professional (1)
- ☐ Online websites (e.g., NHS) (2)
- ☐ Friends or family (3)
- ☐ Online forums (4)
- ☐ Other (please specify) (6) ____
- ☐ None of the above (5)

Display This Question:

If Where did you get information about resolving the issue? = GP/ medical professional

Q11 What advice did your GP/ medical professional offer?

- ○ Medication (e.g., Viagra) (1)
- ○ Lifestyle changes (e.g., stop smoking, lose weight etc.,) (2)
- ○ Other (please specify): (3) ____

Display This Question:

If Where did you get information about resolving the issue? = GP/ medical professional

Q12 Did the GP run any routine tests to identify the cause of ED?

- ○ Yes (mental health assessment) (1)
- ○ Yes (blood test, or medical imaging etc.,) (2)
- ○ No- my doctor did not run any tests (3)

Q13 Was the given advice helpful?

- ○ Yes (1)
- ○ No (2)
- ○ Somewhat (4)

Q14 Which of the following do you think could cause ED?

- ☐ Stress/other mental health conditions (1)
- ☐ Unhealthy diet (2)
- ☐ Obesity (3)
- ☐ Smoking (4)
- ☐ Health conditions (e.g diabetes, heart conditions, high blood pressure, clogged vessels) (5)
- ☐ Certain medications (7)
- ☐ Alcohol or other substance abuse (8)
- ☐ Previous pelvic surgeries or spinal injuries (9)
- ☐ None of the above (16)
- ☐ Other (please specify): (17) ____

© Imperial College of Science, Technology and Medicine

Q15
Which of the following approaches do you think can help to manage ED? (please select one or more)

- ▢ Stress management (e.g breathing techniques, mindfulness, meditation) (1)
- ▢ Diet modification (2)
- ▢ Weight loss (3)
- ▢ Pelvic floor training (19)
- ▢ Weight training/ physical activity (4)
- ▢ Cardiovascular training (18)
- ▢ Reducing alcohol intake (6)
- ▢ Smoking cessation (7)
- ▢ medications (e.g viagra) (8)
- ▢ Vacuum pump training (9)
- ▢ Supplements like L-Arginine (10)
- ▢ Testosterone Replacement Therapy (11)
- ▢ None of the above (16)
- ▢ Other (please specify): (17) ____

Display This Question:

If Have you had issues in getting an erection over an extended period of time (3-6 months)? != Never

Q16

What have you personally done to alleviate ED? (please select one or more)

- ☐ Stress management (e.g breathing techniques, mindfulness, meditation) (1)
- ☐ Diet modification (2)
- ☐ Weight loss (3)
- ☐ Pelvic floor training (18)
- ☐ Weight training/ physical activity (4)
- ☐ Cardiovascular training (19)
- ☐ Reducing alcohol intake (6)
- ☐ Smoking cessation (7)
- ☐ medications (e.g viagra) (8)
- ☐ Vacuum pump training (9)
- ☐ Supplements like L-Arginine (10)
- ☐ Testosterone replacement therapy (11)
- ☐ None of the above (16)
- ☐ Other (please specify): (17) ____

Display This Question:

If Have you had issues in getting an erection over an extended period of time (3-6 months)? != Never

© Imperial College of Science, Technology and Medicine

Q17 Have these selected measures had positive effects on your quality of erections?

○ Significant effect (1)

○ Moderate effect (2)

○ No effect (3)

Q18
There are lifestyle medicine apps (such as Regimen) which work as a solution to improve the quality of erections through lifestyle modifications - would you consider using an app as a standalone therapy or in addition to other treatments to prevent, manage or improve the quality of your erections?

○ Yes (standalone) (1)

○ Yes (in addition to pharmacological treatments) (3)

○ No (2)

---

*

Q19 What is your age?

____

Q20 Are you sexually active?

○ Yes (1)

○ No (2)

Q21 Are you a regular smoker?

- Yes (1)
- No (2)

Q22 How often do you exercise?

- Never (1)
- Less than once a week (2)
- Once a week (3)
- 2-3 times a week (4)
- 4 or more times a week (5)

Q23 Are you overweight?

- Yes (1)
- No (2)

Q24 What best describes your diet?

- Very healthy (1)
- Generally healthy (2)
- Average (3)
- Generally unhealthy (4)
- Very unhealthy (5)

Q25 Do you have a disability?

- Yes (1)
- No (2)
- Prefer not to say (3)

Q26 Do you have any mental health conditions?

- No (6)
- Yes - depression (1)
- Yes - anxiety (2)
- Other mental health conditions (4)
- Prefer not to say (3)

Q27 Do you have any long term health conditions?

- Yes (2)
- No (3)
- Prefer not to say (4)

Display This Question:

If Do you have any long term health conditions? = Yes

Q28 Which of the following long term health conditions do you have? (please select one or more)

- ☐ High blood pressure (1)
- ☐ Cardiovascular disease (2)
- ☐ Diabetes mellitus (3)
- ☐ High blood cholesterol (4)
- ☐ Other (7)

Q29 Are you taking any of the following medications?

- ☐ Antidepressant drugs (for treating depression) (1)
- ☐ Anti-hypertensive drugs (to lower blood pressure) (2)
- ☐ Anti-androgenic drugs (for hair loss/ prostate) (3)
- ☐ No (4)

© Imperial College of Science, Technology and Medicine

Q30 What is your relationship status?

- ○ Married (1)
- ○ Single (2)
- ○ In a domestic relationship (3)
- ○ Divorced (4)
- ○ Widowed (5)
- ○ Other (please specify): (6) ____

Q31 What best describes your ethnic group or background?

- ○ White (2)
- ○ Mixed/Multiple ethnic groups (3)
- ○ White and Black Caribbean (4)
- ○ Asian/Asian British (5)
- ○ British Black/African/Caribbean (6)
- ○ Other (please specify): (7) ____

Q32 We are looking to interview up to 30 participants (via telephone, Skype or Microsoft Teams) to gain a deeper understanding of your experiences and perceptions towards erectile dysfunction. If you are interested in being contacted for an interview (approximately 30 minutes), please enter contact information and we will be in touch; **if you are not interested please click next to register your responses**. All information will be confidential.

- ○ Name: (4) ____
- ○ Email: (5) ____
- ○ Mobile number: (6) ____

End of Block: Default Question Block

© Imperial College of Science, Technology and Medicine
